# The Digital Transformation of Business Model Innovation: A Structured Literature Review

**DOI:** 10.3389/fpsyg.2020.539363

**Published:** 2021-01-07

**Authors:** Selma Vaska, Maurizio Massaro, Ernesto Marco Bagarotto, Francesca Dal Mas

**Affiliations:** ^1^ Department of Management, Ca’ Foscari University of Venice, Venice, Italy; ^2^ Department of Management, Lincoln International Business School, University of Lincoln, Lincoln, United Kingdom

**Keywords:** digital transformation, business model innovation, structured literature review, value creation, value delivery

## Abstract

This paper has a two-fold aim: to analyze the development of the digital transformation field, and to understand the impact of digital technologies on business model innovation (BMI) through a structured review of the literature. The results of this research reveal that the field of digital transformation is still developing, with growing interest from researchers since 2014. Results show a need for research in developing countries and for more collaboration between researchers and practitioners. The review highlights that the field is fragmented among disruptive technologies, shared platforms and ecosystems, and new enabling technologies. We conclude that digital transformation has impacted value creation, delivery, and capture in almost every industry. These impacts have led to the employment of a variety of new business models, such as those for frugal innovation and the circular economy.

## Introduction

The phenomenon of digital transformation (DT) has become very popular in recent years (Fitzgerald et al., 2013; Kane et al., 2015). Digital transformation or “digitalization” is “the integration of digital technologies into business processes” (Liu et al., 2011, p. 1728). The exploitation of digital technologies offers opportunities to integrate products and services across functional, organizational, and geographic boundaries (Sebastian et al., 2017). As a consequence, these digital technologies increase the pace of change and lead to significant transformation in a number of industries (Bharadwaj et al., 2013; Ghezzi et al., 2015), since they have the “power” to disrupt the *status quo* and can be used to drive technological change (Bharadwaj et al., 2013). Digital technologies have revolutionized the way industries operate (Dal Mas et al., 2020c), introducing the concept of “Industry 4.0” or the “smart factory” (Lasi et al., 2014). Digital platforms have created a new way of operating for companies and organizations in a “business ecosystem” (Presch et al., 2020), which has led to changing dynamics in value networks (Gray et al., 2013). Digital technologies have substantially transformed the business (Ng and Wakenshaw, 2017) and society, bringing fundamental changes through the new emerging approaches of the circular and sharing economy.

For strategy researchers, the three characteristics of digital technologies, namely, digital artifacts, digital platforms, and digital infrastructures (Nambisan, 2017) create opportunities for a layered modular architecture and present to firms the strategic choice of following a digital innovation strategy (Yoo et al., 2010). This has drastically changed the nature of strategizing, since many digitized products offer new features and functions by integrating digital components into physical products (digital artifacts), and can simultaneously be a product and a platform (with related ecosystem). In this regard, the literature has coined the term “platfirms” to define those companies relying their business models (BMs) on a web platform (Presch et al., 2020). Moreover, digital infrastructures like data analytics, cloud computing, and three-dimensional (3D) printing are providing new tools for rapid scaling (Huang et al., 2017). Therefore, digitalization blurs the boundaries between technology and management, providing new tools and concepts of the digital environment that are changing dramatically the way firms face new managerial challenges, innovate, develop relationships, and conduct business (Verma et al., 2012; Bresciani et al., 2018).

The new digital environment requires firms to use digital technologies and platforms for data collection, integration, and utilization, to adapt to platform economy (Petrakaki et al., 2018) and to find growth opportunities to remain competitive (Subramanian et al., 2011). Besides, recent research shows that firms utilize external venturing modes (e.g., startup programs and accelerators; Bagnoli et al., 2020) to develop dynamic capabilities (Enkel and Sagmeister, 2020). Digitalization is therefore seen as an entrepreneurial process (Henfridsson and Yoo, 2014; Autio et al., 2018) where firms in pursuit of digital transformation render formerly successful BMs obsolete (Tongur and Engwall, 2014; Kiel et al., 2017) by implementing business model innovation (BMI), which is revolutionizing many industries. Indeed, the literature suggests that in designing an appropriate BM, it can be possible to benefit from the potential embedded value in innovation (Chesbrough and Rosenbloom, 2002; Björkdahl, 2009). For instance, firms adopting digital technologies consider data streams to be of paramount importance and assign to them a central role in supporting their digital transformation strategies (Zott et al., 2011), in contrast to traditional BMs frameworks (Pigni et al., 2016). For this reason, digital technologies inherently link to strategic changes in BMs (Sebastian et al., 2017) and consequently, the development of new BMs (Hess et al., 2016).

In the digital context, BMs have become a new unit of analysis (Zott et al., 2011) to examine the changing effects of digital technologies on the way firms produce and deliver value through BMI. As the literature suggests, BMI provides opportunities in capturing profits in a system of networked activities (Zott and Amit, 2010; Amit and Zott, 2012), and in enhancing firm performance (Foss and Saebi, 2017). The role of the BM is essential in identifying the crucial aspects behind a digital strategy. Indeed, it helps firms in applying the digital lens to innovate their BM to create an appropriate new value (Berman, 2012). However, this process is still evolving (Ferreira et al., 2019) and many questions remain unanswered for entrepreneurs and managers, especially in relation to the integration of digital transformation strategies and business transformation strategies (Matt et al., 2015), in order to realize the “digital business strategy” (Bharadwaj et al., 2013). Indeed, a recent study (Atluri et al., 2018) argues that digital transformation and the opportunities it creates for BMs in every sector are still in the beginning.

Given the increased interest in investigating the relationship between digital transformation and BMI in academia and its importance for practice as well, the purpose of this paper is to understand better what we currently know about the digital transformation of BMI. Specifically, our aim is to review and critique the state of research in the digital transformation of BMI literature, provide a comprehensive, holistic overview of the digital transformation of BMI covering many perspectives, and outline avenues for further research. We adopt Teece (2018) definition of BMs as “mechanisms for creating, delivering, and capturing value” to reflect the value proposition, target segments, value chain organizations, and revenue capture components (Foss and Saebi, 2017). For BMI, we apply the definition by Foss and Saebi (2017): “designed, novel, and non-trivial changes to the key elements of the business model innovation and/or the architecture linking these elements.” According to this definition, BMI involves changes in the individual components and in the overall architecture of the BM.

From a theoretical perspective, this study contributes to these digitally-enabled types of BMIs, which make the emergence of BMs a promising unit of analysis for undertaking innovation strategies. It also responds to the knowledge gap in the literature and enriches our understanding in the digital transformation of BMs (Visnjic et al., 2016). In addition, the results of this study may help practitioners from a variety of industries who seek guidance to understand how digital transformation of BMI can be achieved through value creation and capture (Casadesus-Masanell and Ricart, 2010). This study may help especially practitioners in incumbent firms, since digital transformation of their BMI is a highly complex process requiring a sequence of interdependent strategic decisions (Aspara et al., 2013; Velu and Stiles, 2013).

The paper is organized as follows: the next section explains the method of data collection and analysis used for the structured literature review. This is followed by the results of the study and answering the three research questions addressed in the methodology. The following section focuses on discussing the existing gaps in the literature and avenues for further research. The final section of the paper discusses the conclusions, contribution, and implications for theory and practice.

## Methodology

This paper adopts a structured literature review. According to Massaro et al. (2016), a structured literature review is “a method for studying a corpus of scholarly literature, to develop insights, critical reflections, future research paths, and research questions.” The structured literature review was adopted because “it is based on a positivist, quantitative, and form-oriented content analysis for reviewing literature” (Massaro et al., 2016). This method follows a 10-step process that enables the researcher to “potentially develop more informed and relevant research paths and questions” (Massaro et al., 2016), advancing theory, which is the objective of the literature review (Webster and Watson, 2002).

We wrote a literature review protocol to guide us during the process of reviewing the literature. The protocol-driven approach offers researchers a framework to select, analyze, and assess papers with the aim of ensuring robust and defensible results through reliability and repeatability (Massaro et al., 2016). In the further step, we defined the research questions that aim to bring new insights from the literature review. We identified the following research questions in the protocol document:

RQ1. How has the field of digital transformation developed over time?
RQ2. What is the focus of the literature on the digital transformation of BMI?
RQ3. How has digital transformation facilitated BMI in the literature?

The next step was to determine the type of studies to consider for the review. We decided on the keywords to use to search for articles and the criteria for article selection. Following the keywords used in previous studies in the digital transformation literature, we decided to search using “digital transformation,” “digital disruption,” “technolog* change,” “organis* change,” “disrupt*” and “business model.” As the specific aim of this study is to offer a holistic understanding of the digital transformation of BMI, we purposefully focused on scholarly empirical research that provides insights into how digital transformation is impacting the innovation of BMs. Nodes for coding were determined based on previous systematic literature review (SLR) studies (Massaro et al., 2015; Dal Mas et al., 2019, 2020a). According to these studies, nodes examine information related to authors, the time distribution of publications, country of research, the focus of the paper and methodology. We added nodes about industry sectors, the disciplines of the studies, theories used, and potential impact on the value creation, delivery, and capturing process. These nodes were added to gain deeper insights into the development of the field and suggest implications for further advancement. These nodes were integrated into a framework that served for the coding of the papers and the analysis of the results. The framework, with a description of parameters, is provided in Table 1.

**Table 1:** Classifying framework for literature review.

Parameters	Specifications/variables
Bibliographical/Source-info	
Author	Author demographics
Time distribution of publications	Year article published
Journal titles	Where the article is published
Country/Region of research	Origin of the data
Industry sectors	Empirical setting of the article
Methodology	Computer modeling and simulation
	Conceptual paper
	Explanatory
	Exploratory
	Mixed method
	Special issue
	Viewpoint
	Theoretical viewpoint
Discipline	Economics
	Entrepreneurship
	Finance and accounting
	General management and strategy
	Information systems
	Innovation and technology
	Marketing
	OB and HR
	Operations
	Other
Focus of the paper	Disruptive technologies
	Shared platforms and ecosystems
	New enabling technologies
Theoretical perspectives	Theoretical perspective
	Actor-network theory
	Dynamic capabilities
	Relational view
	Discovery-oriented, theories in use approach
	Grounded theory
	Interpretative cognitive theory
	Value-chain approach
	Digitalization level-servitization
	Business model canvas
	Co-evolutionary perspective
	Portfolio theory
	Not specified
Impacts on value	Digital transformation and value creation
	Digital transformation and value delivery
	Digital transformation and value capture

After identifying the keywords and the framework for the study, we started the collection and selection of papers in a multi-staged process. Firstly, we searched in the Scopus database with the defined keywords in the protocol. This first search revealed 215 publications. In a second step, in order to control the quality of articles, we restricted the search to peer-reviewed journals in the Business and Management category that were ranked 3, 4, and 4* in ABS evaluation. With this additional restriction, we did not take into consideration book chapters, book reviews, and conference articles. In this second search, we, therefore, found articles published in peer-reviewed journals from 1996 to 2020, which reduced the number of publications to 126. After collecting all the articles, each paper was checked for the inclusion of keywords in the title, abstract, and keywords, in order to ensure that the articles fit the research objective of the study. The criteria for article inclusion required the existence of string words about both digital transformation and BMs, which were connected by the Boolean operator AND. When screening publications, we found only a few articles about digital transformation, which were published before 2014. Other articles talked about digital transformation or disruptive technologies, but not about the impact or the connection with BMI. The articles which were not focused on both disruptive technologies and BMI were excluded. At the end of the process, 54 articles were excluded, and the final sample of publications included 72 research articles.

We used the NVivo12 software package for the analysis of the final list of papers. The folder with the selected papers was imported into the software. Each article was coded based on the same nodes as specified in the framework in order to reach the aim of the SLR and avoid researcher bias. We created nodes that were related to the bibliographical information of articles, methodology, discipline, the focus of the paper, and theoretical perspectives. These nodes were used to answer the first two research questions of our study. We created another node for the third research question, to code all the impacts of new enabling technologies on BMI.

After having coded all the papers, following the steps of the protocol, the research group shared the coding project among the members in order to verify that the coding complied with the research questions and the framework of the study and to ensure inter-code reliability. Next, analysis of the dataset developed insights and critique in the field of the digital transformation of BMI. Part of the work in this study was intended to advance the knowledge in the field of digital transformation, by highlighting gaps, identifying new avenues for research, and raising new research questions.

## Results

### RQ1: How Has the Field of Digital Transformation in BMI Developed Over Time?

This section provides an overview of the development in the field of the digital transformation of BMI. It reports the findings related to the descriptive features of this emerging field of research.

#### Author Demographics

The list of analyzed articles shows that there does not seem to be any author domination in the field in terms of the number of publications. Ghezzi and Li are the only authors who published three papers. Several scholars contributed to the research field with two articles each (Bogers, Bose, Frank, Frattini, Gupta, Mangematin, and Wang). All the other authors have published only once in the field of digital transformation of BMI. Most of the articles are co-authored. The analysis of the 198 authors of the 72 publications reveals that most of the articles were written by academic scholars. There are no articles written mainly by practitioners, and collaboration between practitioners and scholars comprised of just a few of the publications. More specifically, these collaborations were carried out in very new topics such as platform-based ecosystems and intelligent goods in closed-loop systems. This implies a close relationship between the research field and practitioners, despite the wide practitioner-academic divide. This divide can result from paywalls in publications, and would be helpful to hold common conferences, encourage more engagement with practitioners, and provide open-access journals to overcome it. Otherwise, the growing divide between academics and practitioners results in field fragmentation, as subgroups will form on both sides of the divide. Greater collaboration between practitioners and academics is thus needed in the future to shape this field of study (Serenko et al., 2010). These demographics also suggest that four authors in this field of research have remained focused on exploring further aspects of BMI driven by digital transformation. For instance, Ghezzi published about strategy making and BM design in dynamic contexts in 2015 in Technological Forecasting and Social Change, and in 2017, he published in the Journal of Business Research. This trend of republishing after 2 years in a different journal from the first is also demonstrated in articles by Bogers (2016). The lack of specialization by researchers might also fragment the field further. In the future, more scholars should remain focused on further exploring other aspects of digital transformation impacts on BMI.

#### Time Distribution of Published Articles

**Figure 1:**
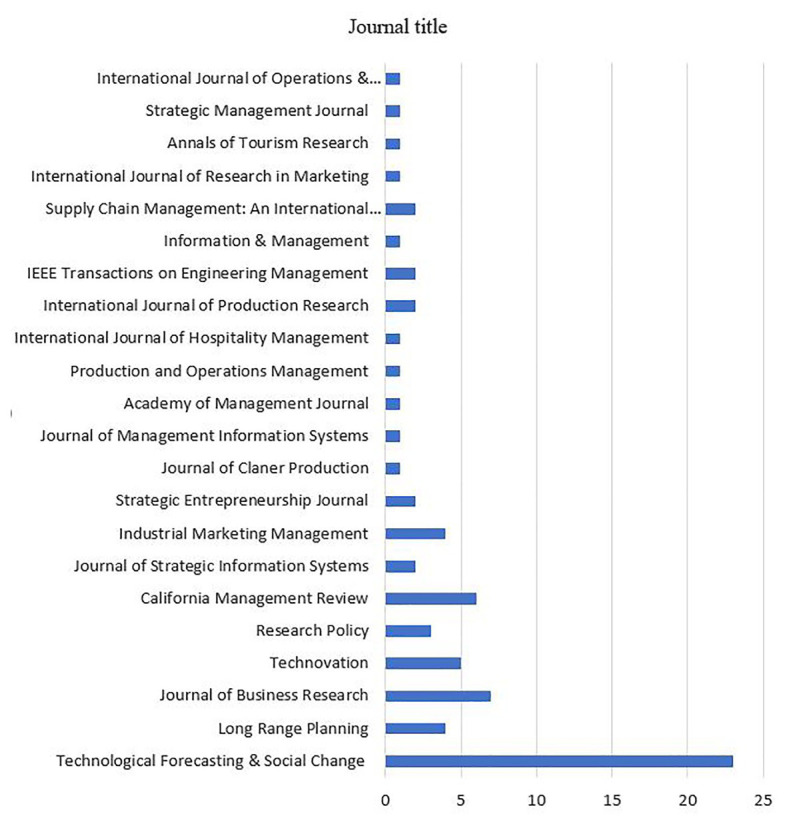
Journals of the selected articles.

The analysis shows that the first article about the digital transformation of BMs was published in 2009. This article was part of a case study of Kodak (Lucas and Goh, 2009), which missed the digital photography revolution when faced by disruptive technology. As can be seen from Figure 1 below, only five papers were published within the next 4 years (until 2013) after the first paper was published. These first papers dealt mostly with a general understanding of the opportunities and barriers created by disruptive technologies on BMI (Chesbrough, 2010), such as, for example, in the case of latecomers that can capture value through a secondary BM (Wu et al., 2010). Publication on the topic remains poor and scattered until 2013 and research continues to highlight the importance of technological discontinuities in the creation of disruptive BMs and the challenge of dominant industry logics (Sabatier et al., 2012). Only Simmons et al. (2013) studied the role of marketing activities in inscribing value on BMI during the commercialization of disruptive digital innovations in industrial projects. Interesting enough, the production of knowledge is particularly active in 2020, which, at the time of the research, saw the articles published in Scopus as of mid-September. Twenty-one meaningful papers were listed in 2020, considering that the year was not finished yet and several more might be in press, forthcoming, or still to be indexed.

In the past 3 years, there has been a growing number of articles published in this field of enquiry, with 42 out of 72 articles published between 2018 and 2020. The greatest interest in publishing about the digital transformation of BMI was recent, where 53 articles (almost 74% of the total sample) were published since 2017. The gradual increase in publications reflects the need to carry out more research in this field, as the impacts and issues related to digital technologies become apparent in many industries. This is shown in articles published during 2014–2015, which try to explore the effects of digitization on incumbent BMs in more depth. Researchers investigated these effects in the publishing industry (Øiestad and Bugge, 2014), and with a special interest in understanding organizational or sectoral lock-ins in creative industries (Mangematin et al., 2014) and the newspaper industry (Rothmann and Koch, 2014). To overcome the challenges of strategy formulation and implementation in dynamic industries, Ghezzi et al. (2015) suggest a framework for strategic making and BM design for disruptive change.

The analysis again reveals the practitioner-led nature of research in this field. As demonstrated above, the time distribution of the articles highlights the relevance of studies in the field. Over time there has been a continuous change in the researched topics, shifting from the impact of disruptive technology on incumbent BMs to the impact of digital technologies on the BMI of digital start-ups. This implies that the field shows characteristics of pragmatic science, where society benefits from the best combination between the relevance of the topic and the rigor of findings (Anderson et al., 2001). The high concentration of the distribution of publications in recent years reveals both the importance of the topic and the increased interest of researchers in this novel field of enquiry. These insights from the analysis of the distribution of articles inform us about the nascent stage this field of enquiry, with rapid growth in 2014. Serenko et al. (2010) consider three indicators to define field maturity: co-authorship patterns, the role of practitioners, and enquiry methods. According to these indicators, we observe that the publication of multi-authored manuscripts increased after 2014, especially in 2016–2017. We further observe more collaboration with practitioners during the 2016–2018 period. In terms of enquiry methods, as a newly emerging scholarly domain, the articles mainly develop theoretical frameworks, revealing the early stage of the field.

Moreover, addressing the topic of the academic-practitioners divide (Bartunek, 2007), the topic seems ideal as an opportunity to gather academics and professionals working together and create some exchange zones to foster a dialog (Romme et al., 2015). While scholars struggle to find robust data to develop sound theories, managers are the ones who see the potential of disruptive digital technologies and their real-world applications, including new BMs.

#### Journal Title

**Figure 2:**
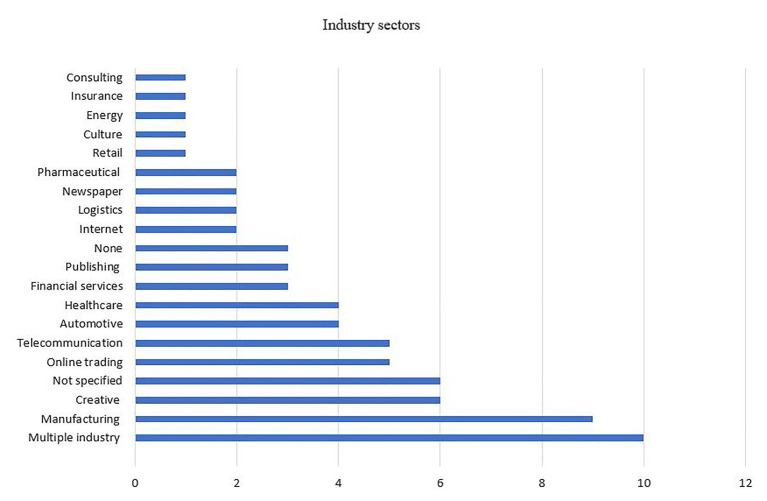
Industry sectors analyzed in the selected articles.

We identified the journals in which these articles were published and their distribution in each journal (Figure 2).

Our analysis shows that a total of 22 journals were captured in this review of literature. The Technological Forecasting & Social Change journal takes the lead for the majority of articles published (23 articles, 32%). The three other journals with a higher number of publications than others are Journal of Business Research, California Management Review, and Technovation. These journals have published seven, six, and five articles, respectively, for a total of 18 articles (25%). The remaining articles were spread over the rest of the journals, and a diverse range of disciplines. This topic seems to be practitioner-led, and with greater relevance recently for businesses, policy makers, and society. This is demonstrated in the Technological Forecasting & Social Change journal, firstly by Sung (2018), suggesting policy implications regarding Industry 4.0 in Korea. Jia et al. (2016) examine the commercialization efforts of a United Kingdom-based 3D printing technology provider to evaluate the financial viability of innovative BMs.

#### Country of Research

**Figure 3:**
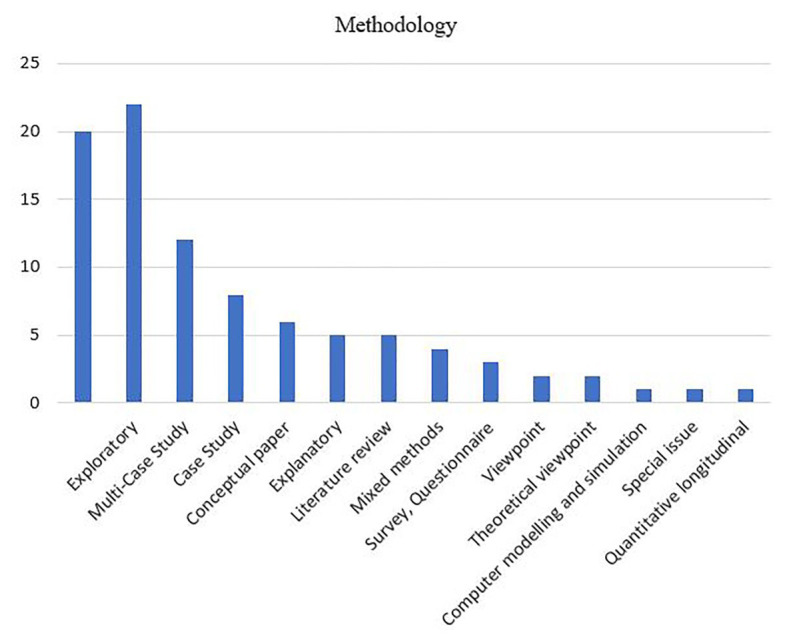
Research methodology of the selected articles.

Part of our analysis was to identify and describe the geographical regions where studies have been conducted. Figure 3 gives a classification of the countries that have been studied in the field of digital transformation of BMI. The left side of the graph includes studies carried out in developed countries, and the right shows developing countries. The results show that most of the research in this field is conducted in developed countries, and within this, the digital transformation of BMI has been studied mostly in the United States and Germany. This concentration of research mainly in these two countries may be the result of governmental efforts, as in the case of German government support for Industry 4.0, or the European Union-funded DIGINOVA digital project for advancing innovation in digital making (Potstada et al., 2016).

According to the analysis, other countries in Europe reflecting the same interest in researchers are the Netherlands, Italy, and the United Kingdom, with two publications in each country (except for the Netherlands, which accounts for three articles). In contrast, emerging and Far-East countries are very under-represented, with China publishing two papers, and India and United Arab Emirates with one article each. This implies that emerging and Far-East countries in general are either ignored or poorly analyzed, despite the presence of several digital firms (let us think about the giant multinational companies like Alibaba, Wechat, or Huawei in China). While there may be publications written in languages different than English or in books or journals not indexed on Scopus, more research is needed in these countries to define the boundaries of theorization in the digital transformation of BMI, which will lead to a better understanding of this phenomenon. As Ghezzi and Cavallo (2020) argue, generalization and the relevance of findings depend on the peculiarity of the context under examination. For this reason, a replication of research in other (mature) contexts should be carried out (Ghezzi and Cavallo, 2020). This will overcome the problem of generalizability with a single geographic region (Simmons et al., 2013).

#### Industry Sectors

**Figure 4:**
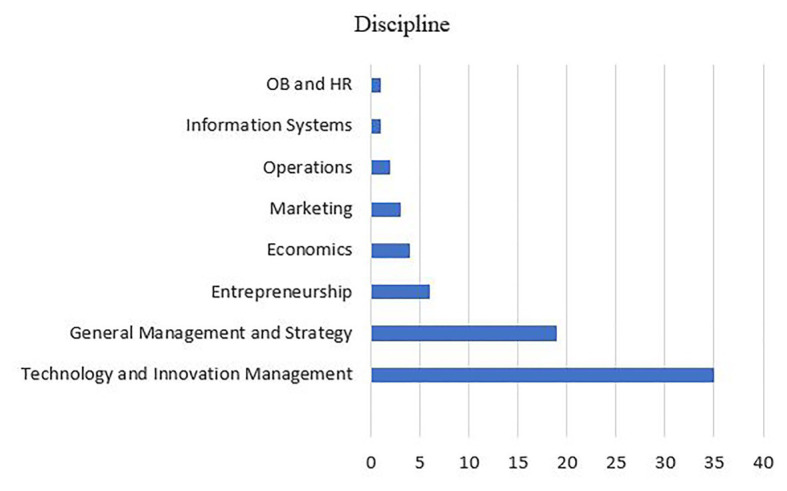
Disciplines of the selected articles.

In order to enhance our understanding of industry influences on the digital transformation of BMI, we classified the articles according to the industry sectors in which their empirical setting was based. As depicted in Figure 4, the articles are based in 18 different specific industries, with several papers referring to multiple sectors together, or not identifying one defined field under investigation.

The results also indicate an almost equal spread of articles among industries, and that there is no concentration in only a handful of industry sectors. Nevertheless, we can identify two groups of industries that are represented by a higher number of articles: manufacturing (nine articles) and creative industries (six articles). A closer examination of these industries shows that the manufacturing industry mainly dealt with consumer goods manufacturing, while creative industry sectors were represented by the accommodation industry and digital game industry. Most remaining articles were spread across the broad range of industry sectors. The focus on only a few industries can be a limitation for the generalization of findings. There is a need to study other industries, such as design, architecture, advertizing, and the fashion industry (Mangematin et al., 2014), which currently do not appear on our list.

#### Research Methods

**Figure 5:**
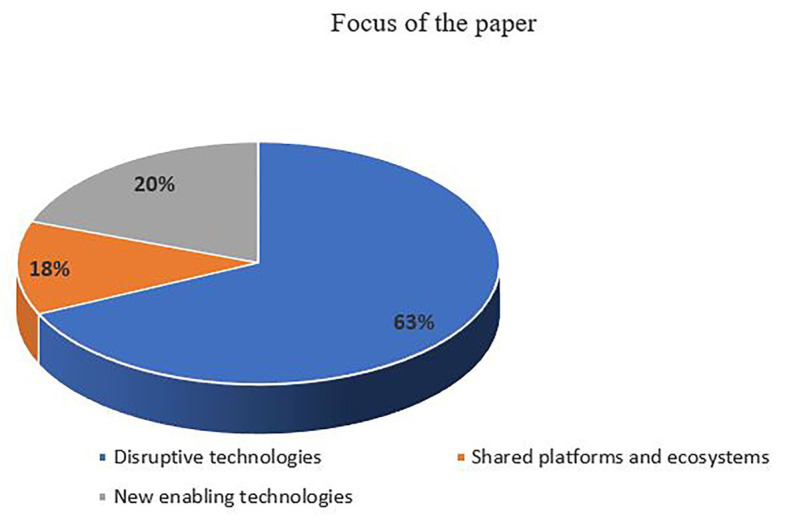
Main focus of the selected articles.

Most studies conducted so far on the digital transformation of BMI have used an exploratory approach (Figure 5).

These studies aimed at achieving a first understanding of the phenomenon of digital transformation of BMI, which is indicated by the extensive use of qualitative research. This finding relates to the fact that digital transformation is a new phenomenon. Consistent with this, Li (2020) argues that we are facing a methodological challenge in the investigation of new emerging trends since these trends “are still at very early stages of development with limited empirical presence”. For this reason, the author suggests using new research methods such as research prototyping and fictional design.

Few longitudinal studies have been carried out. This creates a need for future longitudinal studies, which will help in better understanding the sharing economy and peer-to-peer platforms (Akbar and Tracogna, 2018). The contributions of these studies mainly consist of offering frameworks and propositions derived from explorative research. There have been no further empirical studies to support or refute the suggested propositions. Few papers investigate the relationship between digital transformation and BMI following an explanatory methodology. A considerable number of papers (eight papers) are conceptual or theoretical viewpoints. These insights suggest that the field of research in the digital transformation of BMI has the potential to be restricted to a single paradigm. The absence of positivist research will prevent the wider acceptance and development of the field.

#### Disciplines

**Figure 6:**
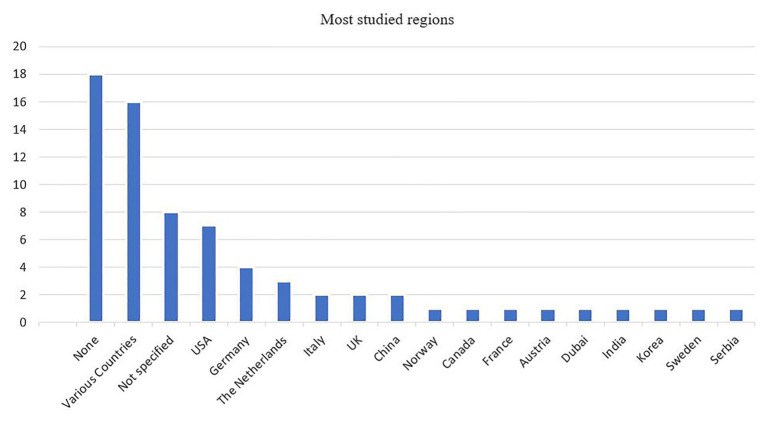
Countries analyzed in the selected articles.

Most of the research is undertaken in the disciplines of technology and innovation management, general management and strategy, and entrepreneurship. Few studies are from the disciplines of economics, information systems, marketing, and operations (Figure 6).

This might primarily be because the purpose of our study is too focused and bridges two different topics: digital transformation and MBI. The other reason might be these three disciplines are more concerned with the impact and implications of the phenomenon of DT. The dominance of only a few disciplines relates also to the journals that are interested in publishing on this topic. Since most of the articles have been published in Technological Forecasting & Social Change, California Management Review, the Journal of Business Research, and Technovation, this affects the disciplines that will be covered by research. The low presentation of articles focusing on operations and entrepreneurship is unexpected, however. This suggests that the field of digital transformation of BMI is fragmented between three major discipline areas, and the predominance of single-discipline research is noted. The fragmentation of the field has implications for the conceptualization and research methodology for the progression of the digital transformation of the BMI field.

### RQ2: What Is the Focus of the Literature on the Digital Transformation of BMI?

#### Main Focus

The literature on digital transformation is dispersed between disruptive technologies, shared platforms and ecosystems, and new enabling technologies such as Big Data, the Internet of Things (IoT), Industry 4.0, Cloud computing, and digital fabrication (DF). Disruptive technologies in the literature refer to technologies that have the potential to introduce new product attributes, which could become a source of competitive advantage (Christensen, 1997); while a platform is defined as “any combination of hardware and software that provides standards, interfaces, and rules that enable and allow providers of complements to add value and interact with each other and/or other users” (Teece, 2018). Taken together, the platform innovator(s) and complementors constitute an ecosystem (Teece, 2018).

The majority of research in this field (49 articles, 63%) has focused on understanding the impacts that new disruptive technologies have on industries, identifying the areas of transformation in activities, processes, and BMs. Only few articles focus on understanding how the process of transformation takes place by drawing on different disciplines and theories.

An analysis of articles about disruptive technologies reveals that in earlier years, the literature (2009–2010) was focused on the challenges and opportunities created for incumbent BMs by these technologies. Some of the articles focus on the challenges faced by incumbents when managing radical technological change. As Chesbrough (2010) notes, there are many “opportunities and barriers in business model innovations” from technological advances. For instance, the case study of Kodak identified organization structure and culture as playing a crucial role in overcoming core rigidities to create new value from disruptive technologies (Lucas and Goh, 2009). Rothmann and Koch (2014) took a very divergent perspective, showing that the digital transformation of BMI fails when companies follow the same old strategic patterns and remain path-dependent. From 2013, focus shifted to ways to overcome these challenges. For example, Karimi and Walter (2016) argue that the adoption of a disruptive BM requires firms to give groups autonomy and allow risk-taking and proactiveness. Kapoor and Klueter (2013) suggested overcoming a firm’s inertia associated with prevailing incumbent BMs by investing in research and development through alliances and acquisitions.

Nevertheless, disruptive technologies bring opportunities to firms who understand how environmental changes necessitate BM modifications. Wirtz et al. (2010) argue that the Web 2.0 phenomenon, based on social networking, interaction orientation, user-added value, and customization/personalization serves as a value offering to traditional internet-based BMs (content, commerce, context, and connection). Another opportunity considered in the literature relates to the introduction of disruptive technologies from advanced economies into emerging economies through a second BMI by latecomer firms (Wu et al., 2010). Firms can also use different tactics (compensating, enhancing, and coupling) to reconfigure their value propositions (Bohnsack and Pinkse, 2017). Table 2 summarizes the challenges and opportunities of disruptive technologies, according to some of the contributions analyzed.

**Table 2:** Challenges and opportunities of disruptive technologies.

Author	Opportunity	Challenge
Lucas and Goh (2009)		Organization structure and culture
Kapoor and Klueter (2013)		Overcoming firms’ inertia associated with prevailing incumbent business models
Wirtz et al. (2010)	Web 2.0 serves as a value offering for traditional Internet business models	
Wu et al. (2010)	Second business model innovation by latecomer firms	
Bohnsack and Pinkse (2017)	Compensating, enhancing and coupling tactics to reconfigure value propositions	

**Figure 7:**
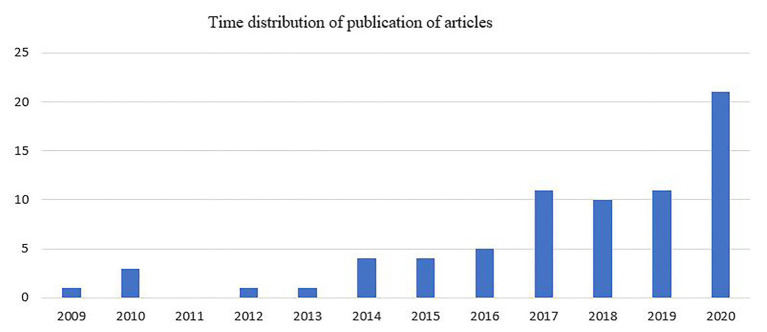
Time distribution of the selected articles.

The second most important topic analyzed, as shown in Figure 7, focused on shared platforms or “platfirms” and ecosystems as new BMs for digital enterprises. Table 3 below summarizes the focus of some of these studies and their findings. We can see that shared platforms and ecosystems are a very recent focus, studied between 2017 and 2018, however, we note that the literature has addressed a number of broad issues which relate to an initial understanding of platforms, starting with their classification into five typologies (Muñoz and Cohen, 2017), and the investigation of the role played by platforms in dealing with disruption (Alberti-Alhtaybat et al., 2019) and BMI (Gupta and Bose, 2019a). Our results show that there is an important focus on financial aspects of platforms and ecosystems. For instance, Teece (2018) and Helfat and Raubitschek (2018) focus on aspects of profiting from innovation, while Khuntia et al. (2017) consider the relationship between the evolution of service offerings and the financial viability of platforms. Analysis of the data also indicates a focus on the managerial issues and success factors of these digital platforms. Since digital enterprises operate in a highly dynamic environment, lean startup approaches (LSAs) have been studied within the strategic agility context. LSAs can be employed as agile methods to enable digital entrepreneurs to innovate BMs (Ghezzi and Cavallo, 2020). Piscicelli et al. (2018) identified the success factors of sharing platforms: the identification of a significant market friction, building of a critical mass of users before implementing a correct pricing level and structure, addressing the hurdles of competition and regulation, and positive interaction fostered between users.

**Table 3:** Focus of literature on shared platforms and ecosystems.

Author (year)	Aim of the study	Results
Muñoz and Cohen (2017)	Typologies of sharing business models	Crowd-based tech business models, collaborative consumption business model, business-to-crowd business model, space-based business model (low-tech), and Utopian sharing outlier business model
Alberti-Alhtaybat et al. (2019)	Dealing with disruption	Building a unique business model based on technological innovations and agility
Gupta and Bose (2019a)	Business model transformation in pioneering digital firms	Technological affordances help companies to strategically learn to adapt to operating environment
Piscicelli et al. (2018)	Success factors for P2P goods-sharing platforms	Business model design and execution; and Ability to experiment and innovate business model
Ghezzi and Cavallo (2020)	Lean startup approaches (LSA) and BMI in digital startups	LSAs are agile methods for BMI for digital startups under conditions of environmental dynamism
Khuntia et al. (2017)	Influence of service offerings evolution in operational maturity and financial viability of Health Information Exchanges (HIE)	Shifting over time from transaction fees, to subscription or hybrid revenue based models
Helfat and Raubitschek (2018)	Profiting from innovation in digital platform-based ecosystems	Innovation, scanning/sensing, and integrative capabilities
Teece (2018)	Profiting from innovation in the digital economy	Understanding of relevant complements, good BM design, and supportive governmental policy
Kamalaldin et al. (2020)	Profiting from digital servitization	Understanding the relational components that can create value
Khanagha et al. (2020)	Profiting from innovation in the digital economy	Understanding the contribution of platforms to competitive advantage

The results shown in Figure 7 indicate that research is also led by recent arising interest in big data (Urbinati et al., 2018), cloud computing (Nieuwenhuis et al., 2018), and closed-loop systems in the circular economy (Rajala et al., 2018). These new enabling technologies allow firms to apply new BMs in support of sustainability issues. The growing intelligence of goods generates novel BMs, which rely on the intelligence of ecosystems within the activities for resources, by shaping closed-loop systems (Rajala et al., 2018). Firms are also engaging more in frugal innovations, allowing them to carry out resource-constrained innovations for emerging markets (Winterhalter et al., 2017).

To conclude, this section develops insights regarding the focus of the literature. The literature that is focused on disruptive technologies advances disruptive innovation theory by proposing culture, organizational structure, and cognitive leadership intentions as important factors affecting company responses to disruptive innovation. However, there is still a missing link in understanding the moderating role of disruptive technologies, based on their digital infrastructure and this requires more research into the conditions and the extent of BM transformations (Gupta and Bose, 2019a). The literature also shows that shared platforms and ecosystems, as well as new enabling technologies, are a very recent focus. In contrast to articles about disruptive technologies that focus on challenges and opportunities, articles about shared platforms consider a broad number of issues from typologies to managerial and financial aspects. Nevertheless, the results show that few articles focus on one topic and the focus shifts quickly, leaving topics under-investigated. This finding highlights the need for more research on topics that are under-investigated and represented by only a few studies. The scattered nature of the field might affect the accumulation of knowledge, as studies do not focus on previous findings.

#### Theoretical Perspectives

Theory development is essential for the proper advancement of knowledge in any field of research (Kuhn, 1970). To develop a better understanding of theoretical perspectives in the field of digital transformation of BMI, we analyzed the articles and determined whether a theoretical perspective was apparent in each. We further analyzed articles that reflected theoretical perspectives and identified whether the theory was an existing one or a new theory. The results of this analysis revealed that the majority of articles (47 articles, 65%) was not based on any discernible theory.

Of the articles with an apparent theoretical perspective, we observed that the majority had adopted theoretical perspectives. Recent contributions (e.g., Vendrell-Herrero et al., 2017; Akbar and Tracogna, 2018; Helfat and Raubitschek, 2018; Teece, 2018) have started questioning and seeking more theoretical frameworks in order to explain and understand the digital transformation of BMI. Interestingly, disruptive innovation theory (Christensen, 1997) was the most popular with five contributions, and other theories were adopted only by single studies. The theory of disruptive innovation was initiated by Christensen (1997) to explain the replacement process of a mainstream innovation by innovations that are cheaper than those on the market and of inferior performance. In this dominant view within the field, which originates from a technological and innovation management perspective, DT is studied at an organizational and individual level of analysis. These researchers incorporate disruptive innovation theory in their studies to show how value generated from technology can be accelerated. For instance, the case study of Kodak (Lucas and Goh, 2009) recognizes culture and organizational structure as crucial elements in creating new value when disruptive technologies are introduced in an industry. Osiyevskyy and Dewald (2015) concentrate on the strategic decisions of managers and argue that responding to ongoing disruption with experimentation depends on a leader’s explorative intentions.

More recent articles that relate the digital transformation of BMI to disruption theory concern topics based on managerial practices of inspiring and managing disruptive innovations in digital entrepreneurships, such as collaborative open foresight (Wiener et al., 2018) and knowledge management (Alberti-Alhtaybat et al., 2019). As Alberti-Alhtaybat et al. (2019) note about the logistic company Aramex that “current study seeks to illustrate their approach to logistics and their mindset regarding disruptive technologies, which is reflected in their particular business model.” Also, for instance, Wiener et al. (2018) argue for collaborative open foresight as a new managerial solution for inspiring disruptive innovations.

We highlight other theoretical perspectives that provide a variety of perspectives on the digital transformation of BMs. Simmons (2013) takes an actor-network perspective to demonstrate that the digital transformation of BMI is a social process facilitated by the negotiation between the network of partners involved. Other researchers use different theoretical perspectives to understand DT of BMI. Akbar and Tracogna (2018) develop their research on transaction cost economics theory to explain the impact of transaction features on the emergence of sharing platforms. Teece (2018) and Helfat and Raubitschek (2018) ground their profit from innovation framework on dynamic capabilities theory. Teece (2018) builds on the recent importance of digital platforms, standards, appropriate regimes, complementary assets, and technologies to show that the mobilization of relevant resources and platform capabilities is an important dynamic ability in managing complements in the ecosystem in order to capture value from it. Similarly, Helfat and Raubitschek (2018) suggest that integrative capabilities are important for designing and orchestrating the alignment of activities and their products with other partners in the ecosystem BMs. Finally, Gupta and Bose (2019a) identify the factors impacting digital transformation of BMs based on affordances theory and attempt to develop a theory of strategic learning for digital ventures, as digital technologies offer firms the potential to develop strategic learning while they adapt continuously to their operating environment. Interestingly, more recent papers (Gupta and Bose, 2019b; Trabucchi et al., 2019) rely on the business model canvas framework (Osterwalder and Pigneur, 2012) to analyze in-depth the variables of innovation, which lead to competitive advantage and communication with the external stakeholders.

These findings suggest that the digital transformation of BMI was firstly related to disruptive innovation theory in the literature and that recently this trend is appearing again. The only difference is that while previous research addresses digital transformation as an extension of the disruptive theory that brings challenges and opportunities to the BM of incumbents, considering digital transformation a consequence of disruptive innovation, recent research relies on disruptive theory and is more focused on practices and methods to manage and inspire disruptive innovations.

To conclude, these theoretical insights suggest that digital transformation has brought a new conceptualization of BMs and new ways for value creation and capture. According to the transaction cost theory, sharing platforms are dominating as BMs, where the transactions between the parties have resulted in the creation of ecosystems. The creation of ecosystems and sharing platforms has pushed research into disruptive innovation theory to emphasize the commercializing value of disruptive technologies. Simons’ article brings a new perspective to our understanding of digital transformation in companies, taking into consideration the moderating role of social aspects in creating value from digital transformation at a firm level. Further research should investigate which social aspects in the network of actors make more contributions to value creation. We also lack an understanding of how the social relationships of the actors in a network contribute value delivery and capture. This perspective of actor-network theory can be very helpful in studying sharing platforms and ecosystems, outside the boundaries of the firm.

Researchers suggest numerous ways for managing disruptive innovation in ecosystems and among firms – through coordination building (Teece, 2018), the implementation of strategic learning processes and structures (Gupta and Bose, 2019a), involvement in collaborative open foresight projects (Wiener et al., 2018), leveraging strategic partnerships through knowledge management (Alberti-Alhtaybat et al., 2019) and using agile methods that enhance strategic agility (Ghezzi and Cavallo, 2020). The digital transformation thus emphasizes not only competition but also collaboration, closing the gap between stakeholders. Referring also to what we discussed previously in the focus of the literature section, digital transformation is enabling companies to work toward issues of sustainability by engaging them in circular and sharing economy approaches. BMs have thus become an open tool for everyday changes related to technological improvements and knowledge management concerning stakeholders and sustainability issues. The digital transformation of BMI now includes technological developments, relationships with stakeholders and sustainability issues in its framework. Our analysis, therefore, suggests that the digital transformation of BMI is a bridge that links the value of strategic innovation management required to solve problems to stakeholders, technology development and sustainability issues, with their opportunities to create and capture value. Further analysis may include the psychological aspects of the various stakeholders, who represent primary actors in the ecosystem, and who may still feature competing interests in the use of digital transformation and its outputs.

This section combines the results of the literature review to understand better the impact of digital technologies on value creation, and the capture and delivery of BMs. In the literature, digital technologies “are regarded to play a critical role in facilitating business model innovations in different sectors” (Li, 2020). New enabling technologies create new ways of doing business for companies and lead to the implementation of new ways of creating, delivering, and capturing value.

#### Digital Transformation and Value Creation

The value creation sub-component of the BM describes the products and services offered to the customer. The review of the literature shows that digital transformation is enabling companies to create new value in a diversity of ways. We identify below four means of value creation and explain each of them.

First, digital transformation allows firms to create new value through the revision and extension of their existing portfolio of products and services. For example, newspaper and book publishing industries adopted a servitization strategy to offer digital products to customers (Øiestad and Bugge, 2014). This extension of products and services relates specifically to the dematerialization of physical products and the switch from product to service logic. In fact, dematerialization and service logic have impacted the pharmaceutical industry through new approaches such as personalized medicine, nanobiotechnology, and systems biology, providing new therapeutic principles in this industry (Sabatier et al., 2012). Other cases in the literature include firms in the retail industry which have created new value by adding a new BMs through online retailing (Kim and Min, 2015).

Secondly, digital transformation enables firms to understand customer needs better and offer new value propositions in accordance with what they want. One type of value proposition creates high personalization with customers. For instance, novel value propositions can provide a high level of involvement for the customers in value co-creation through additive manufacturing and 3D printing technologies, as in the manufacturing industry (Bogers et al., 2016). High-value creations are also based on new BMs that rely fully on recent technological developments such as smart apps, drones, 3D printing, and crowdsourcing delivery to create new value for customers through new services. The adoption of these digital technologies has transformed companies in the logistics industry into technology enterprises, which sell “transportation and logistic solutions without being encumbered by heavy investments in assets” (Alberti-Alhtaybat et al., 2019). In contrast, other value propositions aim to satisfy only the necessary needs. In this case, firms offer new value propositions and even create new markets by addressing the needs of low-income customers in emerging economies (e.g., resource-constraints innovations in the healthcare industry; Winterhalter et al., 2017).

Third, we notice a tendency of some industries, such as financial services, hospitality and automotive services, and healthcare to employ disruptive technologies in their BMs, in order to find solutions for sustainability issues and a sharing economy approach. For instance, the automotive industry is adopting sustainable mobility (Bohnsack and Pinkse, 2017), creating new sources of value by offering a superior product or service (e.g., car-sharing services and mobile applications), or by coupling their products with other services (Bohnsack and Pinkse, 2017). Similarly, embedding the sharing economy approach in the financial services industry is bringing new innovations for processes and services (Gomber et al., 2018), leading to digital banking services, products, and functionality which enhance customer experience (Gomber et al., 2018).

Fourthly, we witness the creation of new value through digital platforms or “platfirms” (Presch et al., 2020) and ecosystems. Digital transformation provides the necessary digital infrastructure for everyone to connect to different actors in networks. For example, in the United States, digital transformation has created new Health Information Exchanges (HIE) organizations, using multi-sided digital platforms to offer information exchange services between different actors in the industry (Khuntia et al., 2017). In the telecommunication industry, the diffusion of data content through mobile devices and the innovation of network infrastructure technology has resulted in a mobile telecommunication ecosystem. In the hotel industry, the emergence of booking platforms (booking.com) and sharing platforms (Airbnb) have brought new value propositions to customers, which are cheaper and more authentic.

#### Digital Transformation and Value Delivery

Value delivery describes the way the activities and processes in a company are employed to deliver the promised value to the customer. The review of the literature reveals a significant change in the way value is delivered in digitally enabled BMs. Digital transformation has challenged core competencies, activities, capabilities, and the roles of firms (Ghezzi et al., 2015; Nucciarelli et al., 2017; Teece, 2018).

Firms are first required to examine their core competences to align themselves with the shift to digital formats and servitization (Øiestad and Bugge, 2014). Their new competencies should include knowledge of digital technologies in order to manage relations with customers efficiently and to use the interactivity of digital channels (Li, 2020). Firms should be open to incorporating new disruptive technologies in order to continuously innovate their operations (Alberti-Alhtaybat et al., 2019).

Second, rapid changes in the new ecosystem business environment introduce the need for new capabilities and more emphasis on specific existing capabilities. New capabilities are necessary to deal with changes in the value chain and ecosystem business environment. For instance, in the pharmaceutical industry, firms need to deploy specific assets and capabilities that relate to the orchestration and management of information flows in the network. Previous literature has highlighted the presence of projects relying on new digital technologies (in that case, the blockchain) to distinguish authentic drugs from fake ones (Dal Mas et al., 2020b). Integrative capabilities help companies capture value in ecosystems and leverage their assets (Helfat and Raubitschek, 2018). In other industries (e.g., telecommunication) marketing capabilities have to deal with decreased costs and technical abilities to deal with changes in the ecosystem. Firms need to be “agile” and leverage platforms and strategic partnerships.

Third, digital transformation implies a change in the activities and processes of the firm. When firms get involved in projects about sustainability, manufacturers in the automotive industry implement environmentally-friendly processes of manufacturing. This undertaking has led companies and suppliers to collaborate on open innovations projects, such as the “Mobility Scenarios for the Year 2030 – Materials and Joining Technologies in Automotive Engineering” (Wiener et al., 2018). The other example involves processes of frugal innovations in the healthcare industry, which are designed to reduce cost in all value chain activities (Winterhalter et al., 2017).

Fourthly, digital transformation has impacted the role of firms in the industry. The shift in the role of actors in the industry results from the entrance of new players. For example, the entrance of new players (web companies) in the telecommunication industry affects value delivery (Ghezzi et al., 2015).

#### Digital Transformation and Value Capture

The value capture of the BM involves the revenue model and its financial viability by focusing on revenue streams and cost structures. The literature review suggests that digital transformation creates various new for firms to decrease costs and increase revenue.

Firms capture value by new enabling technologies. Big data provide companies with the means to reduce uncertainty in decision-making (Urbinati et al., 2018) and to optimize processes and increase the efficiency and quality of products and services (Loebbecke and Picot, 2015). These attributes help firms identify new sources of value in other markets and to reduce the costs of adopting BMs over time.

Firms can capture value from superior value propositions. This is demonstrated in industries such as logistics where customers pay for superior service and solutions, or resource-constraint innovations, for the superior quality of a service network. In the pharmaceutical sector, firms capture value through new value propositions for which companies deliver service to patients. In creative industries, premium prices are based on the exclusivity and personalization level of the service offered (Li, 2020).

Digital transformation allows firms to capture value on platforms by leveraging new technologies and improved customer intimacy (Gomber et al., 2018). Research shows that value capture is influenced by the advancement of services provided, however, and transaction-based revenue models are not appropriate revenue models for achieving viability over time.

#### Future Research Avenues

Based on the results of our literature review, in this section, we discuss the gaps identified in the literature and suggest future research avenues that are relevant for theorizing. We suggest future research avenues, following the previously identified impacts of digital transformation on the new ways of creating, delivering, and capturing value.

##### Future Research Into Value Creation

Research is needed into understanding how companies should manage the trade-off between the cannibalization of existing products and investing in new advanced services for their customers. It remains unclear how companies can develop numerous value propositions for customers that are personalized and always require the co-existence of existing products and product-centric services. The impacts that adding or extending of BMs have on existing BMs are unclear.

It is essential for the manufacturing industry to understand how manufacturers can manage the customization of products and control the value co-creation process with customers (Bogers et al., 2016). In this avenue of research, it would be necessary to consider also the impact of future technological development on value co-creation; for example, how the combination of digital fabrication and Web 2.0 would create new means of value co-creation.

Further research is needed to identify how new BMs emerge, and how value creation is formed in the creative industries, by researching the different interactions among, for instance, crowdfunding platforms, entrepreneurs, and the crowd. There is a lack of knowledge about the effects that crowdfunding platforms have on value creation activities. It would be useful to understand how the collaborative and competitive dynamics of crowdfunding platforms create value for firms.

It remains unclear how agile practices can help firms to create value from digital technologies and customized services. Future research should also consider the application of agile practices in traditional industries. As firms in traditional industries in the context of ecosystems need to carry out more innovation with other firms, this opens an avenue for further research on how agile practices could become a source of value creation.

There is a need for much more research on understanding the role of single technologies such as the Internet of Things, Cloud computing, artificial intelligence, big data, and the blockchain. The application of these technologies in practice will bring direct knowledge for understanding the dynamics of value creation processes as a source of competitive advantage.

Value creation should also be studied regarding how to create value by generating content from customer data. There is still a call for further research into how firms should exploit all this information through analytics that will help them to design better value propositions for customers, according to their needs.

Value creation for customers should also be analyzed stressing the psychological impacts. New insights and inputs come, for instance, from the healthcare sector in dealing with the recent COVID-19 pandemic, with terminal patients relying only on telemedicine to get in touch with their dear ones (Ritchey et al., 2020; Wakam et al., 2020), fostering new possible BMs for firms operating in that field.

Another avenue for further research is to define the boundary conditions under which BMs should be innovated, how often, and how this will impact value creation. Firms learn from the intense and continuous interaction with the high dynamism of the environment and need to undertake changes in the BMI. However, there is still a lack of research defining the boundary conditions driven from the technological advancements that impact value creation in the BMI.

Lastly, it is important to understand the role of new technologies in sustainable issues. It is still unclear how to create new value in the circular economy and from industries where sustainability plays a crucial role, for example, in the retail industry. The link between digital transformation and pro-environmental behaviors of customers, especially from a psychological perspective, appears as a pretty new and promising stream of research (Yusliza et al., 2020).

##### Future Research Into Value Delivery

There is a need for more research on ecosystems. The recent review shows how roles and interdependencies in the ecosystem change remain unclear. New activities, roles, and capabilities should be identified to enhance our understanding of how firms should orchestrate the new relationships in the ecosystem. Knowing how to develop the abilities to manage the delivery network is essential for key players.

The culture shift to advanced servitization requires more research. This is especially necessary for manufacturing companies that now provide digitally advanced services instead of products. This kind of mental shift is difficult for employees and remains a challenge for companies regarding how its delivery network should be organized. The cultural shift is especially important for distribution channels that call for digital servitization.

More research is also needed on understanding the new capabilities required for manufacturing firms that are involved in digital fabrication. More simulation studies should be carried out to better understand how supply chains will be designed for 3D printing.

There should be more research into identifying the role each technology has in enabling firms with new capabilities and roles. These results will offer a clear idea of the technology they should invest and how it should then be related to new capabilities. The attitude toward the use of technologies has been considered by the literature as a soft skill, rather than a technical one (Massaro et al., 2013; Dal Mas et al., 2021; Lepeley, 2021). The open debate concerns how much these skills can be learned, or at least fostered. Further investigation is needed to understand how such skills may be empowered through education in order to facilitate delivery and the translation of knowledge. In this regard, psychological aspects related to the attitude toward new technologies may be taken into consideration, following an interdisciplinary perspective.

##### Future Research on Value Capture

Our results show that investing in digital technologies is costly and undertaking the digital transformation of a firm requires a culture shift. Further studies should investigate how investments in technology relate to the feasibility of revenue models and value capture. Sometimes capturing value from investments in new technologies does not fully exploit the revenue.

Future research should increase our understanding of the value capture of ecosystems, where investments are high. Still, the profits captured by each collaborator actor in the ecosystem are only a fraction of their investment (Teece, 2018).

In the manufacturing industry, the paradigm shift to digital fabrication requires more research into understanding whether value capture is higher for the manufacturer or for the retailer. This can be important in deciding who can invest more in additive manufacturing and 3D printing technologies.

The types of revenue models that should be applied during the evolution of the services are still unclear. There is a need to carry out longitudinal research to explore further the best fit of the revenue models along the lifecycle of the product-centric services (Khuntia et al., 2017).

## Conclusion

This paper uses a structured literature review to provide insights into the development of the field of digital transformation of BMI, to understand the impact of digital transformation on BMI and to provide avenues for further research. The review of the literature shows that the digital transformation of BMI is a new field of research with a growth in interest from researchers since 2014. As there is an increased interest from researchers, we expect a growing number of publications in the field. Our results show that this field of research has no dominating authors, implying that few authors remain focused on exploring further aspects of BMI driven by digital transformation. This hinders the knowledge-building process in the field, as only a few authors make use of prior findings to build cumulative knowledge. Indeed, we observe that topics have shifted over time from a focus on incumbents to digital start-ups and from disruptive technologies to new enabling technologies. This reveals the practitioner-led nature of research in this field, although there is a wide divide between academics and practitioners. For this reason, we suggest more collaboration between academics and practitioners, which will help the field to move from an early stage of maturity toward a mature stage. Collaborations may be facilitated by joint forums, think tanks, interventionist research by academics into firms, publications of the main research results in practitioners’ sources like magazines, financial journals, or internet blog posts.

Our results suggest a need for research in developing and emerging countries, especially those from Asia, as they are significantly under-represented, despite their massive contribution to technological solutions. The manufacturing and creative industries dominate research. This raises the need to study other industries such as design, architecture, advertizing, and the fashion industry (Mangematin et al., 2014) and creating more contents in those sectors, like healthcare, which is relying on DT to cope with the several global challenges, including the recent COVID-19 pandemic (Cobianchi et al., 2020; Dal Mas et al., 2020c; Wang et al., 2020). The extensive use of qualitative methodology also suggests that the potential of the field be restricted to interpretive theory building. This calls for more deductive test theory, which might be found if the field involves more interdisciplinary research in the future.

Our review shows fragmentation of the field between disruptive technologies, shared platforms and ecosystems, and new enabling technologies. The focus of research has been mainly on the understanding of impacts that new disruptive technologies have on industries, identifying the areas of transformation in activities, processes, and BMs. Few studies focus on understanding how the process of transformation takes place by drawing on different disciplines and theories. These insights reveal the scattered nature of the field and a quick shift of topics, leaving them under-investigated. Future research should, therefore, be based more on previous findings, thus helping with the accumulation of knowledge and the identification not only of practical gaps but also theoretical gaps.

We suggest that digital transformation has brought a new conceptualization of BMs to the value creation and capture mechanisms. The review of articles provides a variety of theoretical perspectives on the digital transformation of BMs. Disruptive innovation theory is the dominant theoretical perspective, based on which we propose that the digital transformation of BMI is a bridge that links the strategic management of a company’s disruptive innovation required to solve problems with stakeholders, technology development, and sustainability issues to their opportunities to create and capture value. There is a need for further research grounded on theoretical perspectives of dynamic capabilities and actor-network theory.

The results of our study show that digital transformation has impacted value creation, delivery, and capture in almost every industry, although some fields are more investigated than others. Digital transformation enables firms to co-create value with customers through customized manufacturing; through the adoption of servitization strategies and extension of the existing portfolio of products and services; the creation of new value through digital platforms and ecosystems; and finally, allows firms to address solutions to sustainability issues and even address the very specific and particular needs of customers to enhance their experiences. These changes in value creation have required companies to examine their competences, roles, activities, and capabilities. Firstly, firms should possess first-hand knowledge of digital technologies to manage relations with customers efficiently. Secondly, firms should be prepared to shift their roles as new players enter the ecosystem. Thirdly, involvement in sustainability projects, frugal innovation, and circular economy requires a change in activities and processes. Fourthly, integrative capabilities have become necessary for firms to deal with changes in the value chain and ecosystem environment. The adoption of new enabling technologies allows firms to reduce uncertainty in decision-making and capture value from improved customer intimacy and superior service.

To advance research on digital transformation of BMI, we also suggest some future avenues with regard to impacts of digital transformation on value creation, delivery and capture. The identification of these theoretical gaps can be argued to help the advancement of literature on the digital transformation of BMI.

Our study has limitations. Firstly, this paper considers only research published in leading journals, listed in the ABS classification with 3, 4, and 4*. This can be a limitation due to missing results published in other journals that might be relevant for the aim of our study. Secondly, there are some implications from the conclusions of this study. The results are valid only for the specific time period we consider in this study, until September 2020. As we previously saw, since research in the field is experiencing high interest and an increasing number of contributions yearly, future research works could modify our findings. The conclusions derived in this research are based on exploratory research, where sometimes a single case study approach is followed (Wiener et al., 2018), or sharing platforms are evolving over time (Piscicelli et al., 2018) and where IT industry is characterized by short innovation cycles (Nieuwenhuis et al., 2018). Nevertheless, this research into the digital transformation of BMI can provide practitioners with new insights about the phenomenon, and will help them to continually innovate their BMs and remain competitive, as new technologies become more ubiquitous.

## Author Contributions

SV and MM conceived the idea of the paper. SV wrote the first draft. EB and FM reviewed and fixed the manuscript. All authors contributed to the article and approved the submitted version.

### Conflict of Interest

The authors declare that the research was conducted in the absence of any commercial or financial relationships that could be construed as a potential conflict of interest.
